# A meta-analysis for vaccine protection rate of duck hepatitis a virus in mainland China in 2009–2021

**DOI:** 10.1186/s12917-023-03744-8

**Published:** 2023-09-29

**Authors:** Lina Ye, Siyu Zhou, Huiling Zhang, Tangjie Zhang, Daiqi Yang, Xingping Hong

**Affiliations:** 1 Institute of Comparative Medicine, College of Veterinary Medicine, Yangzhou University, Yangzhou, 225009 Jiangsu China; 2 Jiangsu Co-innovation Center for Prevention and Control of Important Animal Infectious Diseases and Zoonoses, Yangzhou, 225009 Jiangsu China; 3 Health Pharmacy, New York, NY 10013 USA

**Keywords:** Duck hepatitis a virus, Meta-analysis, Vaccine protection rate, Mainland China, Duck

## Abstract

**Background:**

Duck hepatitis A virus (DHAV) is a single-stranded, positive-strand small RNA virus that causes a very high mortality rate in ducklings. The DHAV-3 subtype incidence rate has recently increased in China, causing great economic losses to the waterfowl breeding industry. We analyzed the protection rate of DHAV vaccines used in mainland China from 2009 to 2021 and evaluated the effectiveness of vaccine prevention and control to reduce the economic losses caused by DHAV to the waterfowl breeding industry. We screened five electronic research databases and obtained 14 studies and patents on the protection efficiency of DHAV-1 and DHAV-3 vaccines.

**Results:**

Meta-analysis demonstrated that immunized ducklings produced higher antibody levels and had a significantly higher survival rate than non-immunized ducklings [relative risk (RR) = 12, 95% confidence interval (CI) 6–26, *P* < 0.01]. The age of the ducks and vaccine valence did not affect protection efficiency. Data source analysis of the vaccine protection rate demonstrated that the vaccines conferred immune protection for ducklings in both small-scale experiments and large-scale clinical conditions. The analysis results revealed that although the vaccines conferred protection, the immune protective effect differed between small-scale experimental conditions and large-scale clinical conditions. This might have been due to non-standard vaccination and environmental factors.

**Conclusions:**

Domestic DHAV vaccines can protect ducklings effectively. The subjects immunized (breeding ducks or ducklings) and vaccine valence had no effect on the protective effect. Both small-scale experiments and large-scale clinical conditions conferred immune protection on ducklings, but vaccine immunization under small-scale experimental conditions had slightly better protective effects than large-scale clinical immunization.

**Supplementary Information:**

The online version contains supplementary material available at 10.1186/s12917-023-03744-8.

## Background

Duck viral hepatitis (DVH) is an acute, highly contagious, and often fatal disease of young ducklings (< 4 weeks old). DVH is caused by duck hepatitis virus (DHV), is characterized by bleeding in the liver, and is often accompanied by neurological symptoms. The clinical manifestations are depressed mood, appetite loss, and often, head and neck inversion, lying on the ground; the mortality rate is very high. Post-mortem examination reveals that the liver is enlarged, greenish, displays distinct ecchymotic hemorrhages, and has a brittle texture. Kidney and splenic swelling may also be evident. Cutaneous hemorrhage is often noted [1].

DHV is the general name of many viruses that can cause DVH. In 2009, the International Committee on Taxonomy of Viruses divided DHV into the small RNA virus, astrovirus, and hepatotropic DNA virus families [2]. Small RNA virus family DHVs are termed duck hepatitis A virus (DHAV) and include DHAV type 1 (DHAV-1), which was first isolated in the United States in the 1950s, the DHAV-2 strain isolated in Taiwan, and DHAV-3, which was identified in China and South Korea [3–6]. The family Astroviridae includes duck astrovirus type 1 (DAst-V1) and DAst-V2 [7, 8]. In China, DVH is mainly caused by DHAV-1, DHAV-3, and DAst-V1. DHAV-2 has not been isolated in mainland China. In the aforementioned regions, many ducks were co-infected with multiple serotypes, usually involving DHAV-1 and DHAV-3 [9, 10].

In addition to biosafety measures, many scientists in mainland China have focused on DHAV vaccine research for many years and have made progress. Live attenuated vaccines for DHAV-1 are commercially available. The A66 attenuated vaccine developed by the Jiangsu Academy of Agricultural Sciences and the CH60 attenuated vaccine developed by the School of Veterinary Medicine of Sichuan Agricultural University have obtained the new veterinary drug registration certificates, and experiments have proved that the vaccines provide good DHAV-1 immune protection for ducks [11, 12]. Zhu [13] injected 2000 LD50 DHAV-3 into ducks immunized with DHAV-1 vaccine under experimental conditions and reported that the DHAV-1 vaccine did not confer protection against DHAV-3. Many researchers have performed DHAV-3 vaccine research and development and have made significant research progress, yielding commercial bivalent vaccines that are a combination of type 1 and 3 vaccines: Li et al. [14] developed a live attenuated vaccine with good immunogenicity after continuous passage of the DHAV-3 YDF strain for 110 generations in chicken embryos. Song et al. [15] constructed a recombinant Lactococcus lactis named NZ3900-VP1 that could express the DHAV-3 VP1 protein. Oral vaccination with L. lactis NZ3900-VP1 significantly induced specific anti-VP1 immunoglobulin G (IgG) antibodies and mucosal secretory IgA (sIgA) of DHAV-3 in mice and ducklings. The ducklings vaccinated with L. lactis NZ3900-VP1 were effectively protected when encountering natural DHAV-3 infection. Niu et al. [16] constructed a bivalent vaccine against duck enteritis hepatitis by inserting the DHAV-1 VP0 gene into duck enteritis virus vector.

A recombinant protein vaccine was successfully constructed by inserting the structural polyprotein precursor gene P1 and the protease gene 3CD into the baculovirus expression system and expressing them in insect cells [17]. Kang et al. [18] developed bivalent live attenuated vaccines (DHV-HSBP100 and AP-04203P100) for DHAV-1 and DHAV-3 and reported that immunized ducklings were protected effectively and rapidly against virulent DHAV-1 and DHAV-3 at 2 or 3 days post-vaccination. These studies presented the possibility for the commercialization of DHAV vaccines with higher protection rates in the future.

China is the world’s largest duck meat producer, where duck breeding areas are spread across several provinces such as Jiangsu, Jiangxi, and Zhejiang. According to the Food and Agriculture Organization of the United Nations Statistical Database (FAO Stat), there were 4.855 billion commercial meat ducks in mainland China in 2020.

Many scholars have made progress in virus strain identification and vaccine research and development, but DHAV remains widespread in mainland China, seriously inhibiting waterfowl breeding industry development and causing great economic losses. In this paper, meta-analysis was used to analyze the protection efficiency of recently developed DHAV vaccines in mainland China, evaluate the DHAV vaccine effectiveness, and explore the factors that affected DHAV prevalence to provide helpful information for preventing and controlling DHAV.

## Results

### Selection process

**Fig. 1:**
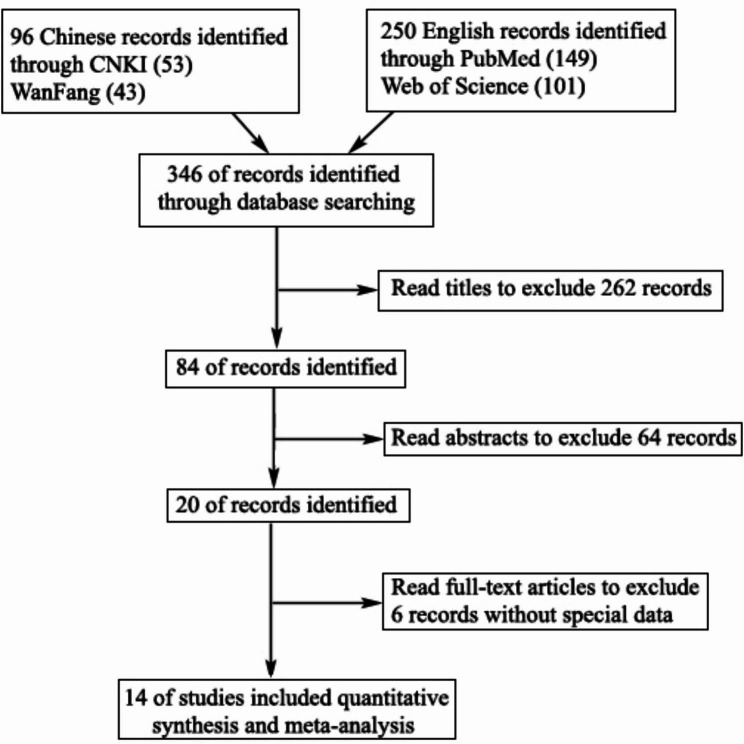
Flow diagram of literature search and selection

We obtained and screened 346 articles. The title, abstract, contents, and exclusion conditions were screened based on the Cochrane manual literature screening process. Eventually, 14 studies were included 13 Chinese studies and one English study [19–32] for meta-analysis of DHAV vaccine protection efficiency. The studies involved a total 950 samples (from experimental and control groups). Figure 1 depicts the flow diagram of the screening and results.

### Data extraction and quality evaluation

**Fig. 2:**
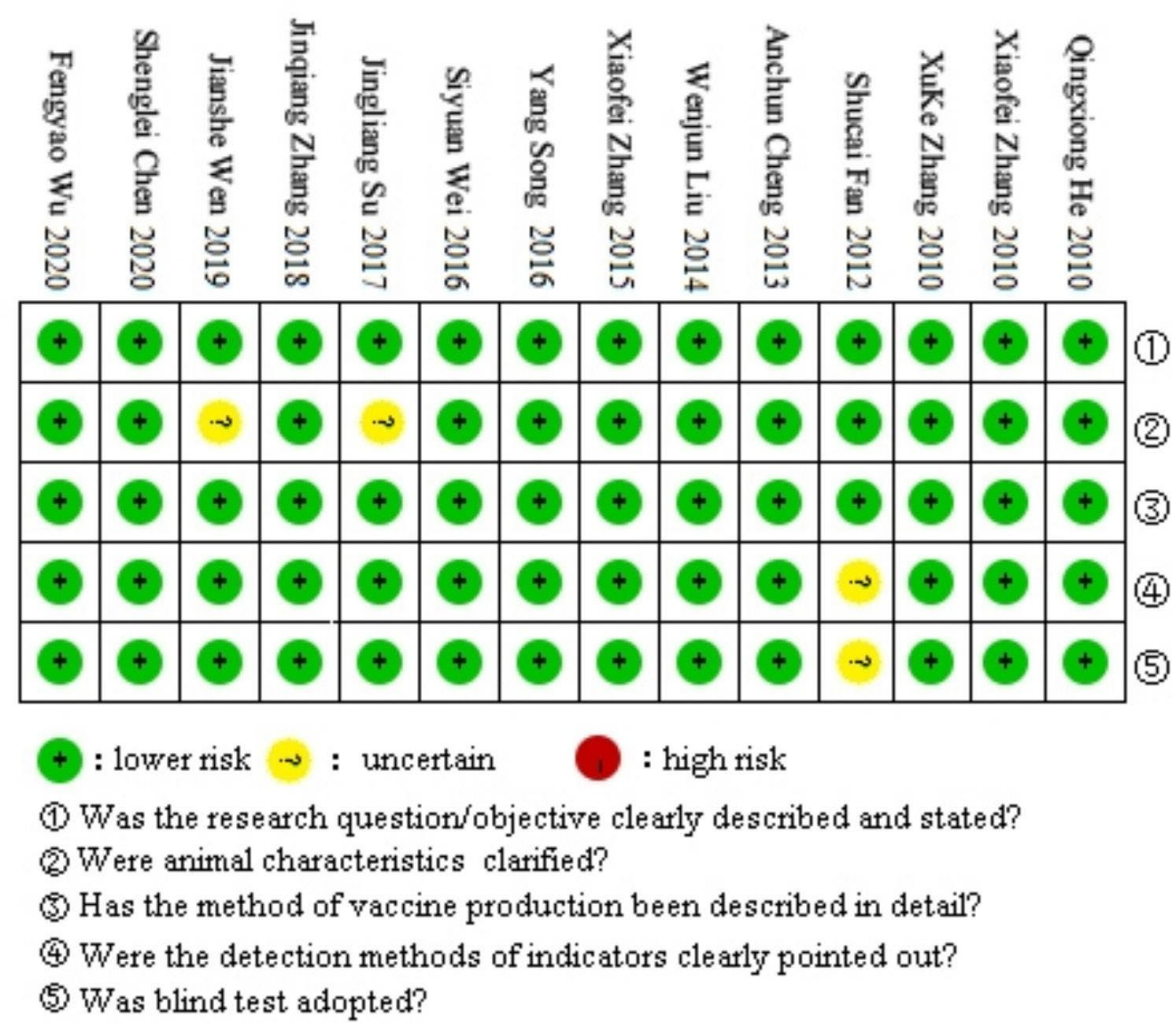
Quality assessment of studies

The first author, publication year, article category, patent number, outcome index, vaccine serotype, and vaccine strain name were extracted from the studies. Table 1 lists the results while Table 2 presents the specific experimental data. The quality of each article was evaluated (Fig. 2) and depicted with the evaluation score [green, low risk (2 points); yellow, uncertain (1 point); red, high risk (0 points)]. The evaluation revealed that the 14 articles met the quality requirements and were included in the statistical analysis.

**Table 1:** Baseline data of the included studies

Author(year)	article category	patent number	Outcome index	vaccine serotype	vaccine strain
Qingxiong He(2010)	Paper	N	Protection efficiency/ serum antibody titer	1	XC-1
Xiaofei Zhang (2010)	Paper	N	Protection efficiency/ serum antibody titer	1	A66
Xuke Zhang (2010)	Patent	201010273200.6	Protection efficiency/ serum antibody titer	1	YC
Shucai Fan (2012)	Patent	201210414017.2	Protection efficiency/ serum antibody titer	1,3	YB3,GD
Anchun Cheng (2013)	Patent	201310011872.3	Protection efficiency/ serum antibody titer	1	CH60
Wenjun Liu (2014)	Patent	201410834435.6	Protection efficiency/ serum antibody titer	1,3	SH,FS
Xiaofei Zhang (2015)	Patent	201510006646.5	Protection efficiency	1,3	HuB60,A66
Yang Song (2016)	Patent	201610781512.5	Protection efficiency/ serum antibody titer	1,3	SD,JS
Siyuan Wei (2016)	Patent	201610285099.3	Protection efficiency	Unstated*	GS14
Jingling Su (2017)	Patent	201710825604.3	Protection efficiency	3	HB80
Jinqiang Zhang (2018)	Paper	N	Protection efficiency	1,3	YB3,GD
Xingjian Wen (2019)	Patent	201910551095.9	Protection efficiency	3	ISA-A117C-C4334A
Shenglei Chen (2020)	Patent	202011299990.5	Protection efficiency	1,3	LSE/QZE
Fengyao Wu(2020)	Paper	N	Protection efficiency	3	SD70

*: Monovalent, but specific vaccine serotype is unstated

**Table 2:** Detailed data of included studies

Author (year)	Vaccine serotype	Data source	Animal immunized	Test Group			Control Group		
				Deaths	Survival	Total	Deaths	Survival	Total
Qingxiong He(2010)	1	①	①	7	3	10	5	0	5
Qingxiong He(2010)	1	①	②	1	9	10	5	0	5
Xiaofei Zhang (2010)	1	①	②	6	94	100	44	6	50
Xuke Zhang (2010)	1	②	②	0	30	30	10	0	10
Xuke Zhang(2010)	1	②	①	2	28	30	10	0	10
Shucai Fan (2012)	1,3	②	①	20	80	100	10	0	10
Anchun Cheng (2013)	1	②	①	0	30	30	30	0	30
Wenjun Liu(2014)	1,3	②	②	2	18	20	20	0	20
Xiaofei Zhang (2015)	3	②	②	2	48	50	4	6	10
Yang Song (2016)	1,3	②	①	0	30	30	10	0	10
Siyuan Wei(2016)	unstated	②	②	0	80	80	15	5	20
Jingling Su (2017)	3	②	②	1	14	15	13	2	15
Jinqiang Zhang (2018)	1,3	①	①	8	52	60	43	17	60
Xingjian Wen (2019)	3	②	②	0	10	10	6	4	10
Shenglei Chen (2020)	1,3	②	②	1	59	60	20	0	20
Fengyao Wu(2020)	3	①	②	0	20	20	9	1	10

Animal immunized: ①: breeding ducks; ②: ducklings.

Data source ①: Clinical trial; ②: patent laboratory trial.

### Data synthesis and analysis

**Fig. 3:**
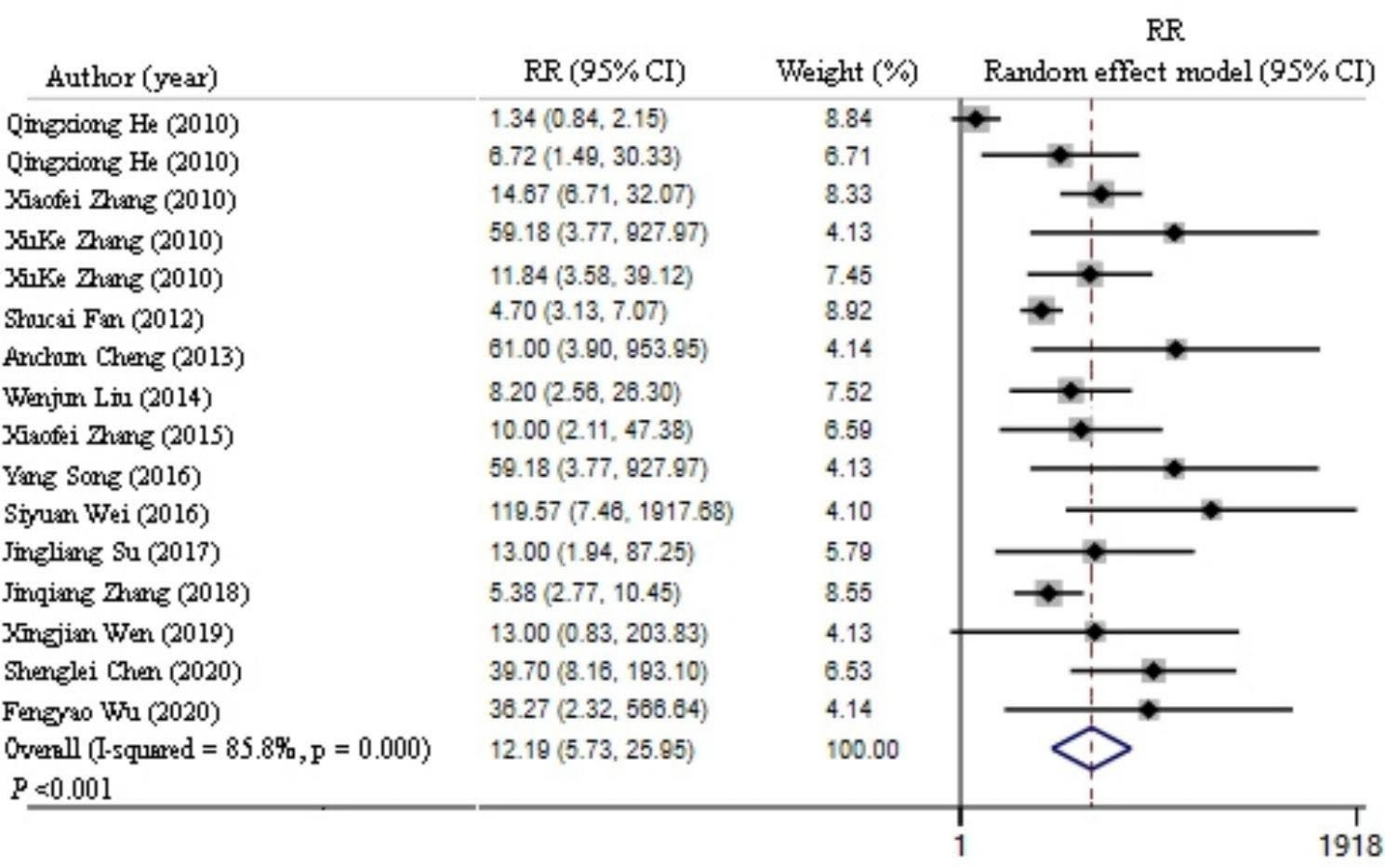
Summary forest plot of protection efficiency of DHAV vaccine

Among the 14 studies, the cohort studies were divided into the control (unvaccinated) and test (vaccinated with the DHAV vaccine) groups (Table 2). The relative risk (RR) was used as the effect scale. The included data were used to test the vaccine protection risk heterogeneity, where I^2^ = 85.8%, which indicated large heterogeneity. Accordingly, the possible sources of heterogeneity were analyzed with the random-effects model. The forest map results of the meta-analysis demonstrated that RR = 12.19 (95% CI 5.73–25.95, *P* < 0.01), where the CI fell on the right side of the invalid line (Fig. 3). The test group had a significantly higher survival rate than the control group.

The vaccine was divided into three groups based on the immune target (ducklings and adult ducks), immune potency (univalent and bivalent), and data source (clinical data and patent laboratory data) for subgroup regression analysis of the reasons for the heterogeneity of DHAV vaccine protection efficiency. The results demonstrated that the regression values of all three groups were *P* > 0.05, which indicated no statistical difference.

**Fig. 4:**
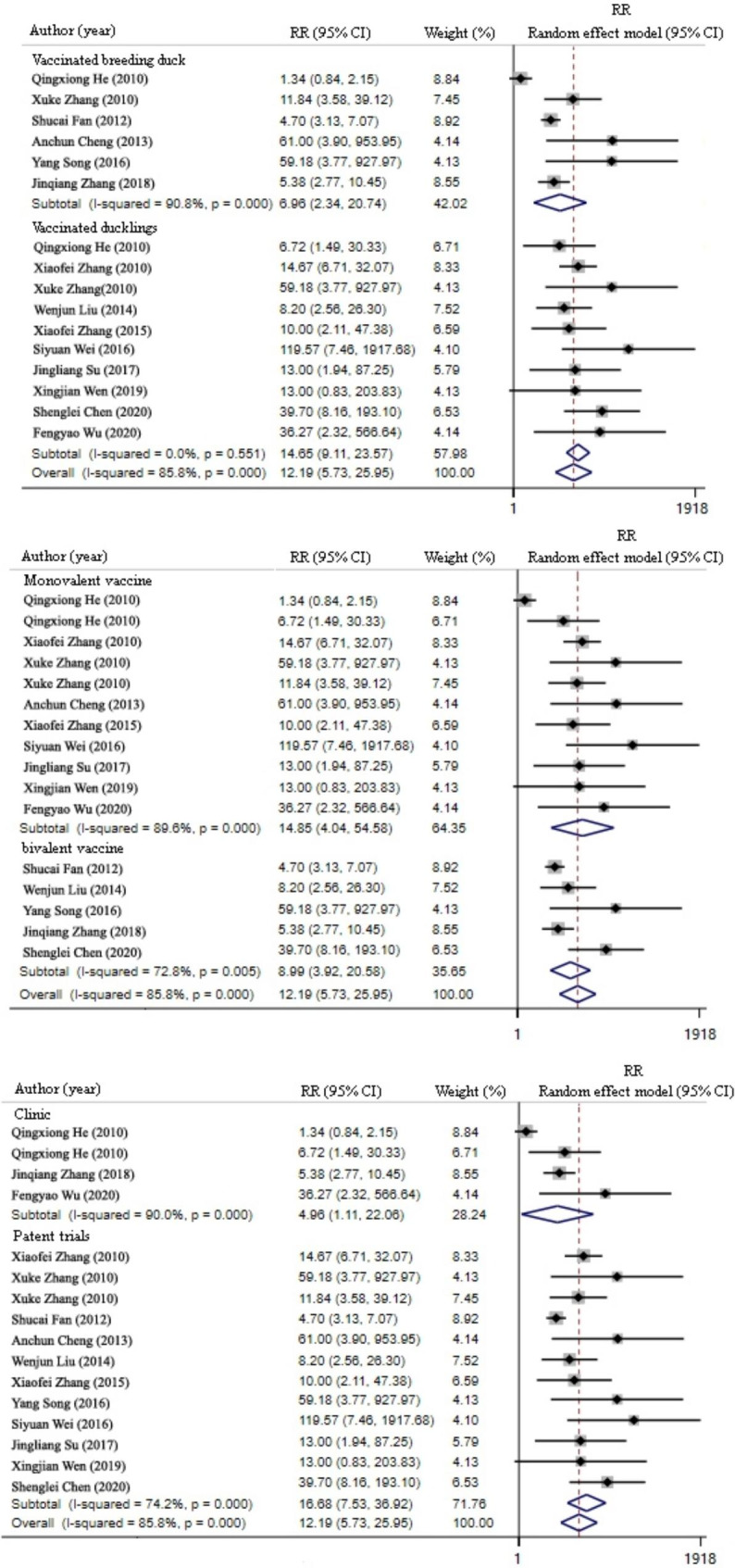
Forest plot of protection efficiency of DHAV vaccine subgroups. Top: immune target groups; middle: vaccine valence groups; C: data source groups

The immune target subgroups were divided into immunized duckling and immunized breeding duck groups. The forest map demonstrated that the CIs of the test groups in the two subgroups fell on the right side of the ineffective line, indicating that the immunized ducklings and breeding ducks had significantly lower RR than the control groups (immunized duckling group: RR = 14.65, 95% CI 9.11–23.57, *P* < 0.01; immunized breeding duck group: RR = 6.96, 95% CI 2.34–20.74, *P* < 0.01) (Fig. 4, top).

The immune valence subgroups were divided into monovalent or bivalent immunization against DHAV-1 or DHAV-3 groups. The forest map demonstrated that the CIs of the test group in the two subgroups fell on the right side of the invalid line, suggesting that the challenged ducklings immunized with the monovalent and bivalent vaccines had significantly lower RR than the non-immunized control group (monovalent immunization group: RR = 14.85, 95% CI 4.04–54.58, *P* < 0.01; bivalent immunization group: RR = 8.99, 95% CI 3.92–20.68, *P* < 0.01) (Fig. 4, middle).

The data source subgroups were divided into clinical trial with large sample size and patent laboratory trial with small sample size groups. The forest map demonstrated that the CIs of the trial groups using vaccines in the two subgroups fell on the right side of the invalid line, suggesting that the ducklings in both trial groups had significantly lower RR than that of the non-immunized control group (clinical trial group: RR = 1.11, 95% CI 4.96–22.06, *P* < 0.01; patent trial group: RR = 16.68, 95% CI 7.53–36.92, *P* < 0.01) (Fig. 4, bottom).

These results indicated domestic DHAV vaccines can protect ducklings effectively. The subjects immunized (breeding ducks or ducklings) and vaccine valence had no effect on the protective effect. Both small-scale experiments and large-scale clinical conditions conferred immune protection on ducklings, but vaccine immunization under small-scale experimental conditions had slightly better protective effects than large-scale clinical immunization.

### Publication bias and sensitivity analyses

**Fig. 5:**
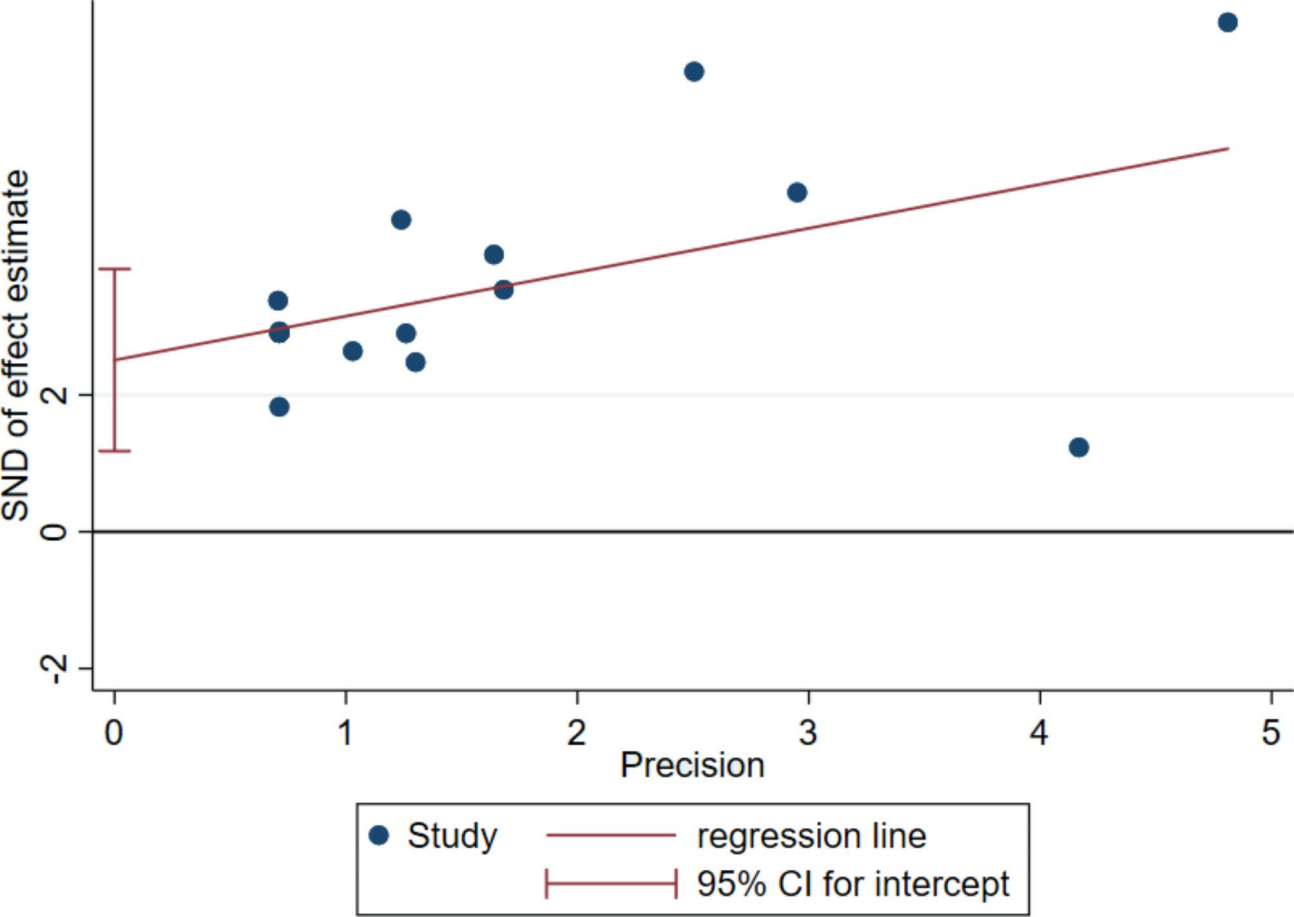
Egger’s test of DHAV vaccine protection efficiency

**Fig. 6:**
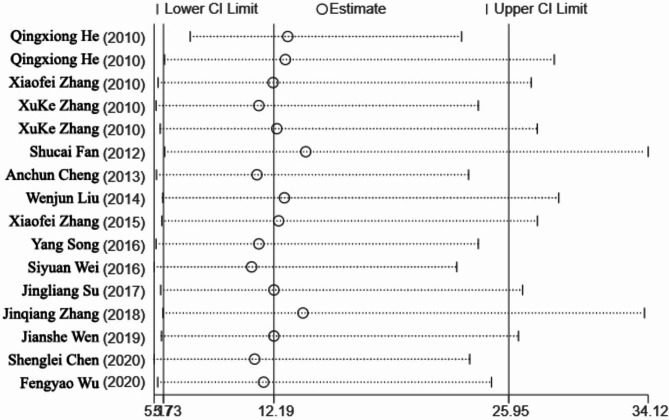
Sensitivity analysis of DHAV vaccine protection efficiency

Potential publication bias was analyzed with Egger’s test, where *P* < 0.01 indicated possible publication bias in the results (Fig. 5). The sensitivity analysis demonstrated that all effect values fell within the 95% CI of the final effect value, which proved that the stability was good and that the test results exerted little influence on the final conclusion (Fig. 6).

## Discussion

We comprehensively evaluated the protective effect of vaccines developed in China in recent years against DHAV in ducks. The 14 articles included revealed a significantly lower risk ratio of DHAV in ducks post-vaccination than in unvaccinated ducks (*P* < 0.01), indicating that the vaccines protected ducks effectively.

As DHAV has been reported in China, Chinese researchers have made produced achievements regarding DHAV vaccines. For example, Yin et al. [33] inoculated duck embryos with type 1 and type 3 DHAV-SH and DHAV-FS strains. After five generations, the embryos were inactivated with formaldehyde to produce bivalent inactivated vaccines. One-day-old ducklings were immunized and 90 ~ 100% of the ducklings were protected after 2–3 weeks. Cheng et al. [19] used a DHAV chicken embryo attenuated virus strain (QL79) and duck plague virus strain to construct a bivalent attenuated vaccine. At 72 h after injection, the neutralization antibody titer against DHAV in the serum was 26.3. Zou et al. [34] constructed a recombinant duck enteritis virus (rC-KCE-2VP1) containing VP1 from DHAV-1 (VP1/DHAV-1) and a VP1 genetically engineered vaccine from DHAV-3 (VP1/DHAV-3). One week after vaccination, virus replication in the ducks was blocked.

Subcutaneous injection of ducklings with the DHAV-1 A66 attenuated strain vaccine for 2–3 days and eye drops for 5 days conferred effective protection against DHAV. After 60 days, the serum neutralization protection titer remained at 26.3. Breeding ducks were generally immunized twice, with a 2–3 week interval. Six months after the second immunization, the antibody neutralization level titer in the serum of the offspring ducklings was 24.8, and their offspring effectively obtained disease resistance [28]. Subgroup analysis demonstrated that regardless of whether the immunized object was breeding ducks or ducklings, immunization based on the standard procedure yielded adequate antibody levels in the ducklings.

The vaccine valence subgroup analysis demonstrated that the ducks injected with DHAV-1 or DHAV-3 univalent vaccine and bivalent vaccine had a significantly lower risk ratio than unvaccinated ducks, indicating good protection efficiency (*P* < 0.01). There was no difference in protection efficiency between the univalent and bivalent vaccines, which proved that vaccine valence was not the key factor affecting protection efficiency. The data source subgroup analysis demonstrated that in either small-scale trial or large-scale clinical conditions, the vaccinated group exposed to the virus environment had lower risk than the unvaccinated group (*P* < 0.01). Nevertheless, the forest map demonstrated that immunization in small-scale tests had significantly lower risk of protective effects than large-scale clinical immunization. It is suggested that the actual clinical application of the developed vaccine differs from the results of small-scale trials. The vaccines for DHAV-3 prevention are actually limited to the lab setting, and there is currently no licensed vaccine for the mass market [32, 35]. So efforts should be taken to develop novel DHAV-3 vaccines [36]. Meanwhile, clinical mass samples should be expanded to test the protective effect of DHAV in clinical cases more objectively and effectively.

Previous studies have shown that variant strains are a factor in the prevalence of DHAV in mainland China. Xiang Meng et al. [37] sequenced and analyzed the entire genome of four isolated DHAV-C strains, and the results suggested that the DHAV-C genome, which has been prevalent in Sichuan in recent years, may genetic recombination of multiple virus strains. Tao Haijing et al. [38] conducted immune protection tests on the isolated wild duck hepatitis virus strain (LY strain) in some areas where immunization against duck hepatitis has failed. The results showed that the antigenicity of some wild DHAV strains in the epidemic area has changed. The DHAV-3 strain DHAV-3 JS found in a duck farm in Jiangsu showed strong virulence [39]. Recent studies have shown that the virulence of DHAV essentially regulates liver damage, and the virulent DHAV may be able to replicate stably in its natural host, while attenuated DHAV cannot [32].

Since 2013, DHAV-3 has gradually become prevalent, and co-infection of these two genotypes is often observed in ducks in mainland China [40, 41]. To prevent DHAV in ducklings, further optimization of the vaccination regimes is needed. The emerging features of DHAV epidemiology should be taken into account in the prevention of the disease. Adult ducks are generally resistant to DHAV, and do not display overt clinical signs after infection [42]. Zhang Yang et al. [43] found DHAV may vertically transmit from breeding ducks to ducklings. Moreover, an epidemiological investigation revealed that many farmers are breeding ducks free-range and lack scientific biosafety measures and appropriate vaccination programmes [44, 45]. Therefore, vaccination programmes should be updated based on the in the disease situation and strain. For example, breeder ducks should be boosted prior to laying, which may decrease virus shedding and vertical transmission. Intramuscular vaccination of ducklings may be considered as effective preventive measure, especially in prevalent areas.

In areas where DHAV at a high level in mainland China, sustained monitoring of duck flocks and the safety of vaccines are also essential for disease control. As previously mentioned, the novel strain infection with high virulence might significantly provide the vaccine failure in the commercial duck farm vaccinated with the first developed strain (DHAV). Infected adult ducks can shed virus and act as the source of infection. Monitoring of duck flocks is recommended. Enough passages of DHAVs in ECEs can lead to significant virus attenuation, but the risk of virulence reversion cannot be totally excluded, especially under the field conditions. Several three attenuated vaccine strains for DHAV-1, including H55, Q50 and C-MLV 85 have been reported to demonstrate enhanced virulence when serially propagated in the susceptible ducklings [46]. So continuous monitoring of vaccine safety is essential.

This article is limited by the fact that, before the data collection deadline, there were no licensed vaccines in China that had been put into practical production and application. The data used in this study was from certain-scale clinical trials and patent disclosure data, resulting in only 14 sets of data being included. Out of these 14 sets, 10 were from patents and lacked the mass market clinical data.

## Conclusion

In this paper, we analyzed the protection rate of DHAV vaccines used in mainland China from 2009 to 2021 and evaluated the effectiveness of vaccine prevention. Meta-analysis demonstrated that immunized ducklings produced higher antibody levels and had a significantly higher survival rate than non-immunized ducklings. The age of the ducks and vaccine valence did not affect protection efficiency. Data source analysis of the vaccine protection rate demonstrated that the vaccines conferred immune protection for ducklings in both small-scale experiments and large-scale clinical conditions. We suggest developing novel vaccines, updating immunization programs, and continuously monitoring viruses to prevent and control DHAV prevail in mainland China.

## Methods

### Data sources and retrieval strategies

The study was conducted according to the Preferred Reporting Items for Systematic Reviews and Meta-Analyses (PRISMA) guidelines. The PRISMA checklist was used to ensure the inclusion of all relevant information in the analysis (Supplementary Data 1). The CNKI, Wanfang, PubMed, Web of Science, and Science Direct databases were searched and the language was limited to Chinese and English. The retrieval interval was from January 2009 to January 2021 and the references included in the study were manually retrieved. The retrieval strategy used a combination of subject words and free words. The data were from the literature and patents. The Chinese search formula was “DHAV” or “duck hepatitis virus”, and “vaccine” while the English search formula was “DHAV” or “duck hepatitis A virus”, and “vaccine”.

### Study eligibility

The data were included if they met the following conditions: (1) research object: DHAV vaccine; (2) subjects: ducks or duck embryos; (3) test method was a randomized controlled trial; (4) evaluation index was mortality or the antibody level; (5) test sample size > 10; and (6) there were exact data on vaccine protection efficiency. If the results were inconsistent, they were resolved by a third party or through negotiation and discussion. Articles that did not meet the above criteria were excluded.

### Data extraction and quality evaluation

The extracted data were the first author, publication type (patent or journal), publication year, patent number, outcome index, vaccine serotype, and vaccine strain name. Microsoft Excel 2016 was used for data management. RevMan 5.3 was used for quality assessment in terms of selection bias, performance bias, detection bias and report bias [47]. The following five items were examined and given a score based on a simple scale: 2 for “yes”, 1 for “unsure” or 0 for “no”.(1) Were the research problems/objectives clearly described and stated? (2) Were the characteristics of the experimental animals clarified? (3) Was the vaccine production method described in detail? (4) Were the relevant index detection methods clearly noted? (5) Was blinding adopted in the measurement? Studies with a total quality evaluation score ≤ 6 were not included in the statistical analysis.

### Statistical analyses

The screened research data were statistically analyzed with Stata 15. Heterogeneity among the studies was estimated by the I^2^ test and then the effect model was selected [48]. A random-effects model was selected if significant heterogeneity among studies was observed (*P* < 0.1 and I^2^ > 50%) according to the Cochrane handbook. The source of heterogeneity was analyzed with meta-regression [49]. Otherwise, a fixed-effects model was used [50]. All effective quantities were expressed by 95% confidence intervals (CI). *P* < 0.05 defined statistical significance.

### Bias and sensitivity tests

Egger’s test is commonly used to assess potential publication bias in a meta-analysis *via* funnel plot asymmetry. *P* ≥ 0.05 indicates that the risk of publication bias is small and *P* < 0.05 indicates possible publication bias [51, 52]. Sensitivity analysis was performed to assess the consistency and stability of our meta-analysis by systematically excluding one study at a time and recalculating the combined DHAV vaccine risk.

### Electronic supplementary material

Below is the link to the electronic supplementary material.

Supplementary Material 1

## Data Availability

Data available on request from the authors.
